# Exploring Bedroom Usability and Accessibility in Parkinson’s Disease (PD): The Utility of a PD Home Safety Questionnaire and Implications for Adaptations

**DOI:** 10.3389/fneur.2018.00360

**Published:** 2018-05-17

**Authors:** Roongroj Bhidayasiri, Onanong Jitkritsadakul, Jirada Sringean, Thitiporn Jantanapornchai, Nitinan Kantachadvanich, Saisamorn Phumphid, Kamolwan Boonpang, Sarawan Pensook, Nicharee Aungkab, Nobutaka Hattori, K. Ray Chaudhuri

**Affiliations:** ^1^Chulalongkorn Center of Excellence on Parkinson Disease and Related Disorders, Department of Medicine, Faculty of Medicine, Chulalongkorn University, King Chulalongkorn Memorial Hospital, Thai Red Cross Society, Bangkok, Thailand; ^2^Department of Neurology, Juntendo University, Tokyo, Japan; ^3^Siam Cement Group Company Limited, Bangkok, Thailand; ^4^National Parkinson Foundation Centre of Excellence, King’s College Hospital, London, United Kingdom

**Keywords:** Parkinson’s disease, nocturnal symptoms, bedroom adaptation, accessibility, usability

## Abstract

**Background:**

Although bedrooms are identified as a major location for accidents among Parkinson’s disease (PD) patients, there are no studies that specifically evaluate the bedroom environments of PD patients.

**Objective:**

To examine the physical bedroom environment of patients with PD by generating a home safety questionnaire to rate bedroom accessibility and usability specifically for PD patients, and piloting it in a small set of PD patients, to identify environmental barriers and recommend adaptations to reduce accident risks.

**Methods:**

Questionnaire development was based on the concept of Personal (P)-Environmental (E) fit. The P component covers five clinical domains that contribute to a patients’ current state of health, including PD-related motor symptoms, PD-related non-motor symptoms, gait and balance impairments, comorbidities, and limitations on specific activities. The E component focuses on both indoor (bedroom, bathroom, living room, stairs, and kitchen), and outdoor (outdoor area and entrance) areas within a home where PD patients commonly get injured. Total score for the whole questionnaire is 171. A higher score indicates more P-E problems.

**Results:**

Comprehension of questions was tested for content validity with an item-objective congruence index of above 0.6 for all items. High internal consistency (reliability) was confirmed by Cronbach’s alpha coefficient of 0.828 (*r*). The pilot in five PD patients gave a mean total score of 48.2 ± 7.29 with a mean score on personal and environmental components of 16.8 ± 5.12 and 31.4 ± 4.51, respectively.

**Conclusion:**

This PD home safety questionnaire is a valid and reliable instrument for examining P-E problems by a multidisciplinary team during their home visits. More studies, involving a large number of PD patients, are needed to establish its utility as a screening instrument in PD patients to assess for home adaptations.

## Introduction

Injuries are common among patients with Parkinson’s disease (PD), frequently occurring at home and most likely in bedrooms (30%), but also in living areas (19%), kitchens (15%), and gardens (14%) (1). Most injuries at home occur as a result of falls in the morning when patients are likely to be in the “off” state, and lose their balance while attempting to get out of bed or during transfers from bed to chair, or get out of bed and trip while walking over a carpet or different surface to their bedroom toilet (2, 3). These two scenarios demonstrate well that imbalance in PD can be due to either motor fluctuation from the disease itself or physical barriers (uneven floor surface). It is not only intrinsic factors (e.g., PD itself, lower extremity weakness, and vision impairment) that contribute to falls in PD, but home hazards, particularly in the bedroom, which are contributing factors in home-associated injuries. As PD progresses, environmental limitations often exceed the functional capacity of the individual patient, resulting in a mismatch between the personal component (P), due to the disease itself, and environmental (E) barriers, mostly located in the patient’s own home (4). P-E fit problems have been shown to contribute to falls in PD patients and are associated with negative health outcomes (5).

In a recent systematic review of home environmental adaptation (HEA) in PD, very few publications were dedicated to the study of this area, and were limited by small numbers of subjects, cross-sectional design without prospective cohorts, and a lack of specific instruments for evaluating P-E fit in PD patients (6). Despite the limited evidence, PD patients were found to have more P-E fit problems than controls, requiring significantly more adaptations in the area of personal care (including bedroom) than control subjects, becoming less independent with more functional limitations (4, 7). In the most recent study, balance problems and inappropriate use of walking devices were identified as major contributors to housing accessibility problems that seem to worsen as the disease advances (8).

While bedrooms were identified as a major location for accidents among PD patients, there are no studies that specifically evaluate bedroom environments in PD patients. As most PD patients spend approximately one-third of each day in their own bedrooms, it is surprising that intervention studies on bedroom adaptations, that could potentially lessen the risk of falling, are virtually non-existent. However, the benefits of bedroom adaptations, including installation of night lights and bed height adjustment, in addition to other home modifications and training, have been observed in frail older adults (not reported to have PD) with a 37% reduction in fall rate when compared to the period prior to the intervention (9). In reality, it seems that what happens in bedrooms is regarded as private and patients or families seem reticent to share the difficulties that patients experience in their bedroom during the night with their physicians (10). This assumption is supported by the fact that very few PD patients (9%) are referred to therapists who are qualified to perform home safety assessments and recommend modifications (11). The lack of referrals is despite patient’s perception that nighttime motor disabilities are the most difficult symptom to improve with current medications; thus, reinforcing an urgent need for studies that reveal the challenges patients experience in their own bedrooms and possible interventions (12, 13). Therefore, the aim of our study is to examine the bedroom environment of patients with PD by generating a scored questionnaire to rate bedroom accessibility and usability specifically for PD patients, and piloting this scale in a small set of PD patients to identify environmental barriers and propose recommendations for adaptations. In this study, we focus our detailed analysis on the bedroom by providing a descriptive analysis of these locations for future adaptations.

## Methods

### Concept and Development of the PD Home Safety Questionnaire

The PD home safety questionnaire was developed at the Chulalongkorn Centre of Excellence for Parkinson’s Disease and Related Disorders (Chulapd, www.chulapd.org) to determine P-E fit among PD patients who may be at risk of injury in their own homes (Data Sheet S1 in Supplementary Material). The questionnaire items were generated by Chulapd multidisciplinary team (MDT) members, consisting of two movement disorder neurologists (Roongroj Bhidayasiri and Onanong Jitkritsadakul), two PD nurses (Nitinan Kantachadvanich and Kamolwan Boonpang), one physical therapist (Nicharee Aungkab), and two architects who specialize in geriatric housing (Thitiporn Jantanapornchai and Sarawan Pensook), to cover personal and environmental components related to PD. K. Ray Chaudhuri and Nobutaka Hattori independently reviewed questionnaire items and provided comments. All members are bilingual and all health-care professionals have extensive experience, of at least 5 years, in the care of PD patients. The development of a PD home safety questionnaire is based on the P-E fit concept originally proposed by the Housing Enabler (HE) investigators when examining the impact of personal limitations on the accessibility and usability of a patient’s home environment (14). While accessibility is a relative concept, describing the encounter between the individual’s functional capacity and environmental barriers, usability generally denotes the ability of a person to move around, be in, and use an environment on equal terms with other individuals (15). Therefore, the PD home safety questionnaire is constructed to have items representing both the personal component, related to PD, and the environmental component, incorporating the physical barriers in a patient’s own home that may affect accessibility or usability. Prior to its development, a literature review was conducted to identify existing questionnaires that have been widely used in the field of HEA. Since all identified questionnaires were primarily developed for older people living in the community with chronic disorders, not specifically PD patients, we only selected questionnaires used as part of successful home safety programs with published results in peer-reviewed journals as models for our questionnaire namely the “HE,” the “usability in my home questionnaire,” and the “housing-related control belief questionnaire” (14, 16, 17).

The personal component of our PD home safety questionnaire covers five clinical domains that contribute to a patients’ current state of health, including PD-related motor symptoms, PD-related non-motor symptoms (NMS), gait and balance impairments, comorbidities, and limitations on specific activities (Data Sheet S1 in Supplementary Material). The items under each domain are derived from standardized questionnaires or rating scales. For PD-related motor and NMS domains, items were developed from, and rated in accordance with the motor section of the Unified Parkinson’s Disease Rating Scale (UPDRS) (18), the non-motor scale (19), the mini-mental state examination (MMSE) (20), Hamilton’s depression and anxiety rating scale (21), the Parkinson’s Disease Sleep Scale-revised version (22), and the criteria for orthostatic hypotension as defined by the consensus statement of the American Autonomic Society and the American Academy of Neurology (23). For the gait and balance domain, items were selected from the activity of daily living section of the UPDRS, divided into gait and balance subdomains. Items on comorbidities were categorized into visual symptoms (V), hearing ability (E), joint problems for weight bearing (J), and weight change of either morbid obesity or significant weight loss. Specific activities were selected from the 16-item Activities-specific Balance Scale that has been identified to influence balance confidence in PD patients (3). All items are based on a “yes” or “no” response if patients had experience these symptoms within the past week. Each item was carefully reviewed by members of the MDT and all members had to agree to include each item. In the case of a discrepancy, all members assessed the evidence again and arrived at a consensus. A total of 36 items were included in a final version with each item generating one score. Since the severity of parkinsonian symptoms of each patient may be differentially affected in different parts of the body, the raters are requested to rank the top three most severe symptoms and multiply the score of these symptoms by two to ensure that the score summation reflects the total symptom burden of individual patient. Therefore, for the personal component, the maximal total score is 59 with a higher score indicating more severe symptoms.

For the environmental component, the MDT took into consideration which areas in the home were associated with the commonest occurrence of injury in PD patients, including both indoor (bedroom, bathroom, living room, stairs, and kitchen), and outdoor (outdoor area and entrance) areas (Data Sheet S1 in Supplementary Material). At each location, items were included if, by consensus of the MDT, they are common physical barriers experienced by PD patients. Similar to the personal component, patients are request to provide a “yes” or “no” response if they had encounter this problem within the previous week. Each item is also categorized by whether it is related to accessibility, usability, or if the patient had encountered an injury in this location within the previous week. In this environmental section, there are 8 items for the bedroom, 12 items for the bathroom, 7 items for living room, kitchen, and stairs each, 9 items for outdoor area, and 6 items for entrance, giving a total score of 112. A higher score indicates more physical barriers for the individual patient. Specifically, for the bedroom environment, two items (bed height and lighting) are related to accessibility while the other six items are considered as part of the usability evaluation (service area, handrails, environment for good sleep, path width, floor, and reliance on assisted device).

### Content Validity

Comprehension of all items was tested for content validity by another expert panel (three movement disorder neurologists, two architects with expertise in geriatric housing, and one PD nurse) who were not involved with item generation. The index of item-objective congruence (IOC) was conducted on all questionnaire items, demonstrating a positive content validation with the IOC index of above 0.6 on all items. The questionnaire was then reviewed by another set of health-care practitioners (one general internist, one general neurologist, one nurse practitioner, one physical therapist, and one occupational therapist), who regularly see PD patients and are likely to implement this type of questionnaire, to ensure that they fully understand the instructions and contents. Only minor revisions to the wordings and grammatical corrections were allowed at this stage.

### Reliability (Internal Consistency)

We performed the reliability test by determining Cronbach’s alpha coefficient in five health-care professionals, consisting of one neurologist, one general internist, two nurse practitioners, and one occupational therapist in a single PD patient’s home. Cronbach’s alpha of this questionnaire was 0.828 (*r*), demonstrating high internal consistency.

## Implementation of the PD Home Safety Questionnaire in a Pilot Trial

**Figure 1:**
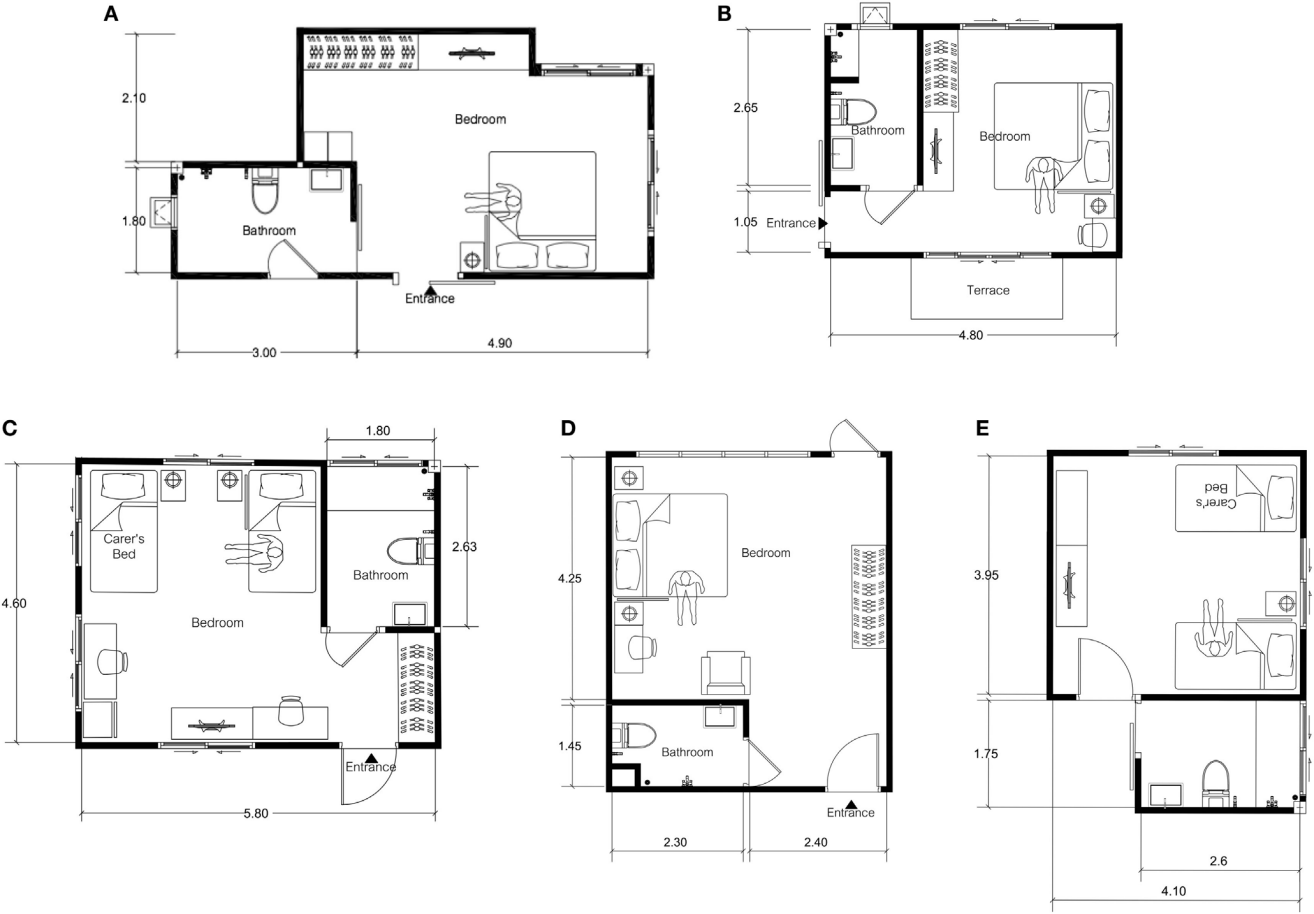
Floor plan of Parkinson’s disease patient’s bedrooms and adjacent bathrooms, labeled as **(A–E)** for patients 1–5 consecutively.

As a pilot trial, the PD home safety questionnaire was employed by another MDT, who was not involved with the item generation and validation process, in five PD homes. Eligibility criteria were the homes of PD patients who had a diagnosis confirmed by a movement disorder neurologist using the standard diagnostic criteria and had been resident in this home for more than 10 years without prior adaptations. All patients must have carers who were their spouses and share the same bedroom environment. All patients were identified by the MDT to have both disabling symptoms from PD and housing problems, particularly in their bedrooms, requiring adaptations. Patients with significant comorbidities were excluded as it would be difficult to establish if environmental barriers occur as a result of PD or other disorders. Due to transport limitations, only those patients who resided in Bangkok were invited to participate. From the eight PD patients who were randomly selected and satisfied the above criteria, three declined the invitation to participate in this pilot trial (response rate = 62.5%). The main reason for refusal was being too embarrassed to show their houses to the MDT. The study was approved by the Ethics Committee of Chulalongkorn University and informed consent was obtained from all participants. Clinical demographics of all five patients were shown in Table 1. Other clinical descriptions and their housing conditions were described as follows (Figures 1A–E).

**Table 1:** Demographic data of five Parkinson’s disease (PD) patients.

Items	Mean ± SD	Min–Max
Age (years)	66.2 ± 6.76	55–73
Disease duration (years)	8.6 ± 3.58	5–13
PD diagnosis	5 (100%)	
Male gender	4 (80%)	
History of falling in the past 1 month	4 (80%)	
Number of urination per night in the past 1 month	3.00 ± 1.73	0–4
Types of assisted device used		
• Cane • Walker • Wheelchair • None	2 (20%) 1 (40%) 1 (20%) 1 (20%)
Number of falls during the past month	13.2 ± 26.20	0–60
Total LED	659.5 ± 462.76	50–1,235
MoCA	26.67 ± 3.51	23–30
MMSE	25.60 ± 4.28	19–30
HY score	3.2 ± 0.83	2–4
Total UPDRS	44.8 ± 16.62	23–69
UPDRS part 1	1.00 ± 1.00	0–2
UPDRS part 2	12.20 ± 6.06	5–19
UPDRS part 3	24.80 ± 7.76	12–33
UPDRS part 4	3.80 ± 1.79	2–6
PDSS-2 score	28.00 ± 9.27	16–40

*LED, levodopa equivalent dose; UPDRS, the Unified Parkinson’s Disease Rating Scale; MoCA, Montreal Cognitive Assessment; MMSE, mini-mental state examination; HY, Hoehn and Yahr stage; PDSS-2, Parkinson’s disease sleep scale-revised version*.

### Patient 1

Patient 1, aged between 55 and 59 years old, has a 9-year history of PD (Figure 1A). Current problems are motor fluctuations with both “on” and “off” period freezing of gait (FOG), particularly when walking through a narrow entrance and during turning. The patient falls once or twice a month, mostly after getting out of bed in the evening to go to the adjacent bathroom. In addition, the patient complains of increasing difficulty in walking upstairs due to leg weakness. The patient lives with a carer who in a two-story house with the bedroom and main bathroom on the second floor. The MMSE score was 27.

### Patient 2

Patient 2, aged between 65 and 70 years old, had a 13-year history of PD (Figure 1B). Due to intractable motor fluctuations, the patient underwent bilateral globus pallidus interna deep brain stimulation 2 years ago with remarkable results in a reduction of dyskinesia and improved “on” periods. However, the balance remains troublesome, particularly during turning in a small bathroom with a tendency to fall backward. The patient falls in the bathroom on almost weekly basis while attempting to turn from the sink to go and sit on a toilet seat. Another common circumstance is when the patient turns in front of the wardrobe. Other complaints include drooling and mumbling speech. The patient lives with a carer in a downstairs bedroom with an adjacent bathroom. The patient was noted to have visual hallucinations in the evening. The MMSE score was 24.

### Patient 3

Patient 3, aged between 55 and 59 years old, has a 5-year history of postural instability and gait dysfunction PD (Figure 1C). Despite levodopa benefits, FOGs remain intractable with numerous fall episodes, mostly in the bedroom while changing clothes in an upright position. The patient also falls frequently when walking from the bed to the adjacent bathroom as the route requires three turns leading to FOG. The left leg was broken last year as a consequence of one of these falling episodes. The patient lives in a one-story house with a carer. The MMSE score was 30.

### Patient 4

Patient 4, aged between 55 and 59 years old, has a 11-year history of PD (Figure 1D). The patient lives in a four-story house with a bedroom and adjacent bathroom located on the second floor. The main problem is frequent “on” period FOG, leading to falls in the early morning when walking from the bed to the toilet. The patient also suffers from nocturnal hypokinesia and early morning off. The patient’s partner who is a carer also has a walking difficulty due to a recent hip replacement. The MMSE score was 28.

### Patient 5

Patient 5, aged between 70 and 75 years old, has a diagnosis of PD dementia for 4 years (Figure 1E). The MMSE score was 19. In addition to cognitive difficulties, the patient reports problems with vision, described as an inability to focus and a lack of depth perception. The carer believes that poor vision contributes to nighttime falls when the patient has 4–5 episodes of nocturia and attempts to get out of bed to go to the bathroom. The patient refuses to wear diapers during the night. The carer finds the situations at home increasingly difficult to cope with.

The administration of the PD home safety questionnaire follows two steps. Step 1: personal component: interview and observation of functional limitations and comorbidities on five domains as described above. All items are dichotomously assessed (yes = 1/no = 0). The interview of this section is led by the neurologist of the MDT with the whole process taking approximately 1 h. Step 2: environmental component, involves the assessment of seven locations of both outdoor and indoor, led by the physical therapist or occupational therapist of the MDT. This step does not involve the PD patient, taking approximately 2 h to complete the process. All statistical analysis was performed using SPSS version 22.0 software (Chicago, IL, USA). A significant level of *p* < 0.05 was set for all statistical tests. Descriptive statistics were performed for all demographic variables expressed as mean ± SD for continuous variables or frequency counts and percentage for categorical variables. Spearman correlation coefficient (*r*) was used to determine the strength of correlation as weak, moderate, or high.

## Results

The results of our pilot trial in five PD patients have demonstrated the utility of the PD home safety questionnaire in capturing functional limitations in both personal and environmental aspects of PD. The demographic data and clinical characteristics of PD patients are shown in Table 1. All five PD patients in this pilot trial experienced their main difficulties in gait and balance with mainly FOG with frequent falls, and mostly located in their bedroom and adjacent bathrooms. The mean total score of the PD home safety questionnaire was 48.2 ± 7.29 with a mean score on personal and environmental components of 16.8 ± 5.12 and 31.4 ± 4.51, respectively (Table 2). With regards to the functional profile in personal component, limitations were demonstrated as a score in each domain of motor, NMS, gait and balance, and ability on specific activities. However, the gait and balance score was relatively high when compared to scores in other domains, consistent with the clinical history, demonstrating the validity of our questionnaire in differentiating the contribution of individual symptom to the whole functional limitations in an individual patient. For the environmental component, we observe a score in all domains, reflecting the presence of environmental barriers in all areas as a result of PD. In the bedroom environment, problems identified were mainly concerned with accessibility, with four out of five PD homes (80%) having bed height higher (55–60 cm) than recommended standard value (45–50 cm) and no supported bed rails available to assist patients when getting out of bed (Table 3). Moreover, two out of four patients (50%) reported minor injuries as a result of bed height problems. Inadequate lighting in the bedroom was also identified in two out of five PD homes (40%) with one patient claiming to have fallen as a consequence. In this pilot group of five PD homes, fewer problems were identified on usability items compared to accessibility items in the bedroom environment (Table 3). One out of the five homes was found to have furniture not secured in its place causing narrow walkways in the bedroom and a slippery floor was identified in another PD home during the MDT visit.

**Table 2:** Results of PD home safety questionnaire in five PD patients.

Scoring	Total score		Min–Max	
Total PD home safety score (171 points)	48.20 ± 7.29		(38–54)	
Personal component score (59 points)	16.80 ± 5.12		(9–23)	
• Motor component (9 points)	3.00 ± 1.73		(0–4)	
• NMS component (24 points)	4.60 ± 1.52		(2–6)	
• Gait and balance score (12 points)	5.40 ± 2.51		(1–7)	
• Comorbidities score (7 points)	0.80 ± 1.09		(0–2)	
• Limitation of specific activities score (7 points)	3.00 ± 1.22		(2–5)	

**Scoring**	**Total score**	**Accessibility score**	**Usability score**	**Injury score**

Environmental component score (112 points)	31.40 ± 4.51 (26–38)	7.80 ± 2.04 (6–11)	11.20 ± 2.77 (7–14)	9.80 ± 4.65 (4–17)
Outdoor				
• Outdoor area (18 points)	6.80 ± 1.92 (5–10)	2.40 ± 1.14 (1–4)	2.40 ± 0.55 (2–3)	2.00 ± 2.12 (0–5)
• Entrance (12 points)	4.20 ± 1.48 (2–6)	2.00 ± 0.71 (1–3)	1.80 ± 1.09 (0–3)	0.40 ± 0.54 (0–1)
Indoor				
• Stairs (14 points)	4.00 ± 0.70 (3–5)	0.40 ± 0.55 (0–1)	2.80 ± 0.84 (2–4)	0.60 ± 0.89 (0–2)
• Living room (14 points)	3.20 ± 1.92 (1–6)	NA	0.20 ± 0.45 (0–1)	2.20 ± 1.48 (0–4)
• Kitchen (14 points)	3.20 ± 1.92 (1–6)	0.60 ± 0.89 (0–2)	0.40 ± 0.54 (0–1)	0.80 ± 0.84 (0–2)
• Bathroom (24 points)	7.00 ± 1.22 (5–8)	1.20 ± 0.84 (0–2)	3.00 ± 1.22 (2–5)	2.60 ± 2.30 (0–6)
• Bedroom (16 points)	3.00 ± 1.41 (1–4)	1.20 ± 0.84 (0–2)	0.60 ± 0.54 (0–1)	1.20 ± 1.30 (0–3)

*Values in parentheses were shown as min–max*.

*PD, Parkinson’s disease; NMS, non-motor symptoms*.

*Total score of PD home safety questionnaire is 171 points. The total scores of personal and environmental components are 59 and 112 points, respectively. The total scores of individual domains in the personal component are as follows: PD-related motor symptoms 9 points, PD-related NMS 24 points, gait and balance impairment 12 points, co-morbidity 7 points, and limitation of specific activities 7 points. The total scores of individual domains in the environmental component are as follows: outdoor area 18 points, entrance 12 points, stairs 14 points, living room 14 points, kitchen 14 points, bathroom 24 points, and bedroom 16 points*.

**Table 3:** Summary score for bedroom assessment in five Parkinson’s disease patients.

Bedroom	Problem with accessibility (yes = 1/no = 0)	Problem with usability (yes = 1/no = 0)	Injury as a result of the problem (yes = 1/no = 0)	Score summary	Remarks
Service area		1	0	1	– Pathway narrower than 90 cm – Furniture is not secured in its place
Handrails		0	0	0	– No handrails installed at necessary location in the room
Environment for good sleep		0	0	0	– Pollutants identified (noise and/or air)
Bed height	4		2	6	– Unsuitable height for bed (lower than 45 cm or higher than 50 cm) – No support bar to assist getting out of bed
Path width		0	0	0	– Less than 0.9 m
Floor		1	1	2	– Slippery floor
Lighting	2		1	3	– Scenario 1: sleep—more than 5 lx – Scenario 2: activity in bedroom—less than 100 lx
Reliance on assisted device		0	0	0	– Assisting person could be interpreted as an assisted device
Score summary	6	2	4	12	

*The score represents the number of bedroom with problems in accessibility, usability, or reported injuries as a consequence*.

*Lux, a standardized unit of measurement of the light illuminance*.

Correlation analysis between personal and environment components of the PD home safety questionnaire revealed a number of significant findings. High and significant correlations were observed between comorbidities and total scores of the questionnaire (*r* = 0.889, *p* = 0.044), and a similar observation was also demonstrated between limitation of specific activities and total score of the questionnaire (*r* = 0.892, *p* = 0.042) (Data Sheet S2 in Supplementary Material). Analysis within the personal component indicated high and significant correlations between motor symptoms as well as gait and balance impairments and total scores of the personal component (motor: *r* = 0.894, *p* = 0.041; gait: *r* = 0.949, *p* = 0.014). For the analysis of the environmental component, high and significant correlation was observed between indoor sum scores and total scores of the environmental component (*r* = 0.975, *p* = 0.005).

### Descriptive Analysis of Bedrooms and Adjacent Bathrooms of Five PD Homes

The descriptive analysis of the bedrooms of the five PD patients was conducted by the MDT, led by the physical therapist and architect who specializes in geriatric housing (Figures 1A–E). In addition to a physical evaluation of bedroom environments, we also interviewed PD patients and their carers on challenges they encountered during the night based on their nighttime activities. As noted from Table 1, nocturia was common with an average of three episodes per night. Most patients experienced FOG on their nighttime trip to the bathroom with reports of inadequate lighting. Common findings are summarized below with proposed recommendations for adaptations.

(1) Beds of all five patients were located too far from the bathrooms for patients to get there and back quickly. Therefore, the beds should be relocated closer to the bathrooms.
(2) Most patients encountered obstacles resulting from the layout of their homes on their walk to the bathrooms. In one home, the patient had to walk through two doorways with three turns to reach his bathroom. Clean and uncluttered spaces make a space feel larger and are important for PD patients who need clear floor space to maneuver in and around the bedroom with a walker or wheelchair or to and from the bathroom at night.
(3) Inadequate lighting was observed in all five bedrooms as all patients used dim lights to promote their sleep. Our recommendation is to install adequate lighting or automatic night lights to help patients find their way during the night.
(4) In three out of five patients (60%), the bed height was found to be 55–60 cm as carers had installed bed risers to help patients get out of bed. If the bed is too high, patients may be at risk of significant injury from falling out of bed. This is a potential risk among PD patients, particularly the ones with parasomnias. Our recommendation is to adjust bed height to be between 45 and 50 cm, which is considered optimal for the elderly population. Bed rails should be installed to prevent patients from falling out of bed and to assist patients with getting out of bed.
(5) Bedroom carpets were found in four bedrooms (80%). They should be removed to prevent tips and falls at night.

## Discussion

This study describes the development of a PD home safety questionnaire, outlining the developmental process under an MDT specializing in PD. The results confirm the validity of the contents and reliability when implemented by an individual member of a multidisciplinary group. The questionnaire was piloted in five PD patients in their own homes, demonstrating its utility in capturing functional limitations as reflected in the personal component and environmental barriers as indicated by the environmental component of this questionnaire. The relatively high scores on gait and balance domain as well as bedroom and bathroom domains are consistent with the clinical histories of all five patients which are dominated by frequent FOG and falls within bedrooms and adjacent bathrooms. Though limited by a small number of subjects, correlation analysis also revealed significant contributions of comorbidities and limitation of specific activities on the total questionnaire score. Motor, gait, and balance symptoms were found to have a significant influence on the personal component score, providing another evidence to support the effect of gait and balance on disease burden (24). Importantly, the significant correlation between indoor scores and the total environmental component scores highlights the important contribution of indoor barriers on the environmental problems faced by PD patients, consistent with prior literature documenting frequent occurrence of indoor injuries among PD patients (1). While our results are exploratory, they provide preliminary, but objective, evidence on environmental barriers that may occur as a result of functional limitations in PD.

When reviewing a patient’s symptom, physicians often focus on the clinical features of individual symptoms, and severity, but ignore on the circumstances or situations where these symptoms occur. A good example is the symptom of FOG where the episodes are often described in relation to a patient’s “on” or “off” periods and whether the patient fall as a consequence, but on many occasions, environmental aspects of FOG episodes are not described. This incomplete information has led physicians focus their treatment of FOG on medications that improve “on” periods that are often found to be insufficient in ameliorating FOG episodes. Looking at environmental perspectives of FOG, there are clearly physical barriers, contributing to repeated occurrence of FOG, including narrow spaces, insufficient lighting, abundance and disorganization of furniture items, home uncleanliness, and clutter. While it seems clear from patient’s descriptions that environment barriers are another important contributing factor to FOG, the therapeutic evidence on environmental adaptations is still lacking, rated as level D (expert opinion), in contrast to several pharmacologic agents receiving a stronger level of evidence (A or B) (25). We propose that each individual symptom of PD, whether it is motor or non-motor, should be reviewed from both personal and environmental perspective so we can ensure that P-E fit is maintained in an individual patient for as long as possible.

While physicians have instruments to evaluate physical symptoms of PD (for example, tremor, gait, or balance), specific instruments for determining environmental barriers among PD patients are still lacking (6). Fortunately, a number of instruments, although originally developed for the elderly in the community without specific disorders, have recently been employed with PD patients. The HE questionnaire, developed for assessment of housing accessibility, is a comprehensive scale based on the notion of P-E fit, taking into account that functional limitations constitute an important component of accessibility problems (26). The HE has recently been studied in over 250 PD patients across all Hoehn and Yahr stages, identifying the significant contribution of balance problems and dependence of walking devices in reducing home accessibility (8). Although the HE has been shown to be a valid and reliable instrument for assessment of housing accessibility, it is a comprehensive instrument requiring special training to administer, and even though it is comprehensive in terms of including all body parts that involve in mobility, the personal items of the HE is not specific for PD symptoms, lacking the contribution of NMS. Therefore, it is doubtful that the HE can be implemented during a PD home visits by an MDT as intended for the PD home safety questionnaire. As a result, we have adopted the concept of P-E fit as proposed by the HE developers, but incorporated specific items of PD symptoms, and simplified the contents so it can be used by a PD MDT during their regular home visits to identify potential areas for home adaptations. Although preliminary, our results acknowledge the significant contribution of balance impairment as one of the main functional limitations, consistent with what has recently been shown with the HE (8), and highlights the bedroom and adjacent bathroom areas of major environmental barriers, supporting previous reports that these areas are common locations for home injuries among PD patients (1).

In recent years, there has been an increasing interest in evaluating the symptoms of PD, treatment responses, and a patient’s daily performance in their own homes, resulting in the development of different types of home battery tests, home monitoring, and remote assessment devices (27, 28). While most devices are developed for specific parkinsonian symptoms or activities (personal component), a specific location within the home has not been considered as a target for adaptations in most published studies. We would like to highlight the importance of the bedroom for wellbeing as we all, not just PD patients, spend approximately one-third of our daily life in this location. In PD patients, the situation is even worse as up to 97% of PD patients experience at least one PD-related nighttime symptoms and treatments for these symptoms are considered unsatisfactorily by most patients (12, 13, 29). The impact of nighttime symptoms on carers has also been demonstrated in terms of increased total carer burden, stress, and poor sleep quality (30). On the environmental side, we are not aware of any published studies that evaluate the efficacy of bedroom and bathroom adaptations for the reduction of injuries (e.g., falls) or the improvement of sleep quality of either patients or their carers. General guidance for bedroom and bathroom modifications are usually provided by professional societies or organizations, but specific recommendations are generally unavailable (31, 32). The data from our pilot study provides a preliminary evidence that physical barriers in the bedrooms of PD patients significantly contribute to P-E fit problems, and should receive a high priority for interventional research.

Our study findings are limited to the validation of PD home safety questionnaire and a pilot trial in a small number of PD patients. Therefore, though it confirms the validity of this questionnaire among PD patients, more studies involving a large number of PD patients are needed to establish its utility as a screening instrument in PD patients who may be candidates for home adaptation. In addition, specific outcomes (e.g., falls at home) should be included in order to determine the sensitivity and specificity as well as a cutoff point for this questionnaire. The time required for full assessment of this questionnaire by a PD MDT is another limitation for implementing it in a routine clinical practice. While the PD home safety questionnaire is developed to enable a full assessment of home environment, the results of our first study focus on the issues of bedroom environment as previously identified as the most common location for PD-associated injuries. Further studies are being planned by our group to implement the PD home safety questionnaire in more subjects to identify specific barriers in other locations as well as the results of adaptations. The strength of our study is the involvement of a PD MDT in the generation and validation of this questionnaire, and that the evaluation was also conducted by a PD MDT in the patient’s own homes. However, it is limited by the lack of control subjects, the small number of PD patients in the pilot trial, and that bedroom and bathroom-related injuries were not clearly defined, for example, the number of falls.

In conclusion, optimal evaluation of PD patients should not be limited to the physical symptoms of the actual patients, but environmental aspects should also be considered as important contributing factors to patient’s safety, quality of life, and wellbeing. Based on the notion of P-E fit, environmental barriers are likely to emerge as the disease advances, necessitating the need for MDT assessment of both personal and environmental contributions to a patient’s functional limitations. In this study, we provide a validated assessment of the PD home safety questionnaire for screening for potential P-E problems by an MDT during their PD home visits. As we all, including PD patients, spend almost one-third of our day in our bedrooms, we propose that the bedroom should receive a priority for HEA research as treatment of individual nighttime symptoms of PD patients, together with appropriate bedroom adaptations, is likely to result in better sleep quality and reduction in nighttime-related injuries for the patients, and decreased burden for their carers.

## Ethics Statement

The protocol was approved by the ethics committee of Chulalongkorn University. All subjects gave written informed consent in accordance with the Declaration of Helsinki.

## Author Contributions

Research project: conception (RB, OJ, and JS), organization (RB, NK, SPensook, and KB), and execution (RB, OJ, JS, NK, SPensook, KB, KC, TJ, NA, and NH). Statistical analysis: design (RB and OJ), execution (RB and OJ), and review and critique (RB and OJ). Manuscript preparation: writing the first draft (RB), review and critique (RB, NH, TJ, and KC).

## Conflict of Interest Statement

TJ and SPensook are employed by Siam Cement Group Company Limited and all other authors declare no competing interests.
