# Supplementary material for: Exploring Bedroom Usability and Accessibility in Parkinson’s Disease (PD): The Utility of a PD Home Safety Questionnaire and Implications for Adaptations
Source: Front Neurol. 2018 May 17;9:360. doi: 10.3389/fneur.2018.00360 (PMC5966531; doi:10.3389/fneur.2018.00360)
Supplement: Supplementary file 1 [file data_sheet_1.docx]

**
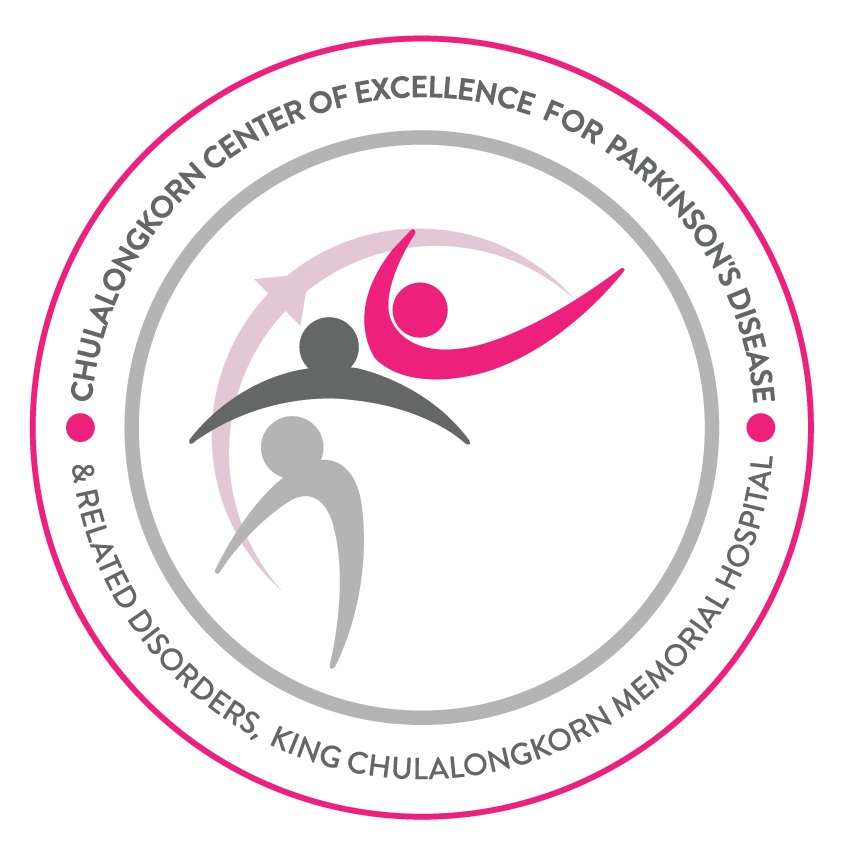
**

**Parkinson’s Disease Home Assessment Questionnaire (PD-Home Safety Questionnaire)**

Total score ranges from 0-171 points

**Instruction:**

The purpose of this questionnaire is to identify potential risk inducing problems patients with Parkinson’s disease (PD) may have in their own homes. The questionnaire is composed of two sections to assess both the personal and environmental impact of PD in the home setting and answers should be based upon how PD symptoms have affected home activities during the previous week. The total score for this questionnaire is 170 points.

1. *Personal component:* consists of 5 domains related to different aspects of parkinsonian symptomatology;
   1. PD-related motor symptoms
   2. PD-related non-motor symptoms
   3. Gait and balance impairments
   4. Comorbidities
   5. Limitation of specific activities
2. *Environmental component*: consists of seven domains determining accessibility and usability of different home areas;
   1. Outdoor: outdoor area
   2. Outdoor: entrance
   3. Indoor: stairs
   4. Indoor: living room
   5. Indoor: kitchen
   6. Indoor: bathroom
   7. Indoor: bedroom
3. In patients with fluctuations who experience ‘off’, and ‘on’ periods, the questionnaire should be performed in each state separately.

***Personal* *component symptom locations:***

The figure below provides anatomical locations for correlation with the patient’s symptomatology. Please rank the three most severe symptoms according to the patient’s complaints or perception of their disability by putting the numbers (1, 2, and 3, where 1 represents the most severe symptom) in the circles provided.


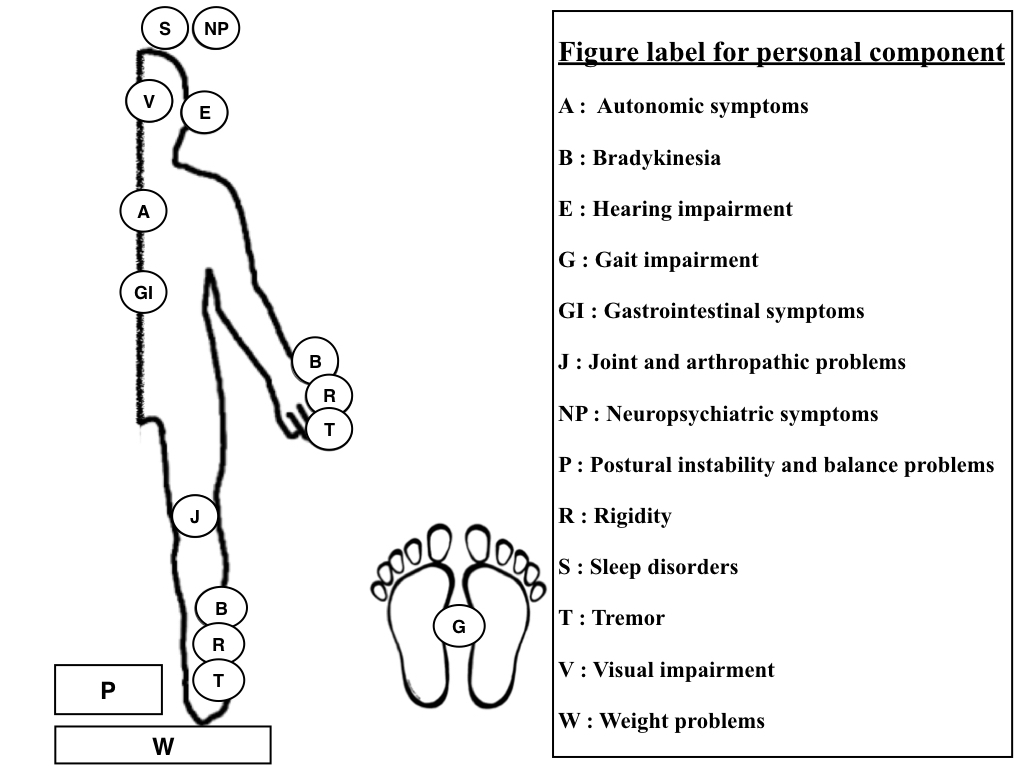


**1) Personal component (total 59 points)**

1.1) PD-related motor symptoms (4 items, maximum score 9 points)

| **Location of the most severe symptoms** | **PD related motor symptoms** | **YES=1 NO=0** | **Multiply the most severe symptoms by 2** | **Remarks (Correlation with standard rating scales)** |
| --- | --- | --- | --- | --- |
| **B** | Bradykinesia |  |  | UPDRS 31: Body bradykinesia and hypokinesia  YES: Presence at least of mild degree of slowness and poverty of movements |
| **T** | Rest tremor |  |  | UPDRS 20: Tremor at rest (upper and lower extremities)  YES: Presence at least of mild amplitude and persistence of tremor |
|  | Action tremor |  |  | UPDRS 21: Action or Postural tremor of hands  YES: Presence at least of moderate amplitude tremor |
| **R** | Rigidity |  |  | UPDRS 22: Rigidity (judged on passive movement of major joints)  YES: Presence at least of mild to moderate rigidity |
|  | Bilateral involvement |  |  | Parkinsonian symptoms involved bilaterally |
| YES; UPDRS scores for each item at least 2 out of 4 | | | | |

1.2) PD-related non-motor symptoms (13 items; maximum score 24 points)

| **Location of the most severe symptoms** | **PD-related non-motor symptoms** | **YES=1 NO=0** | **Multiply the most severe symptoms by 2** | **Remarks** |
| --- | --- | --- | --- | --- |
| **P** | Neuropsychiatric symptoms (P) |  |  |  |
|  | - Dementia |  |  | MMSE of 23 or below |
|  | - Hallucinations, illusion, or delusions |  |  | Presence at least with benign hallucination |
|  | - Depression |  |  | Determined by HAM-D scale > 7 points |
|  | - Anxiety |  |  | Determined by HAM-A scale > 17 points |
| **S** | Sleep disorders (S) |  |  | Determined by PDSS-2 scale |
|  | - REM sleep behavioral disorders |  |  |  |
|  | - Insomnia |  |  |  |
|  | - Sleep disordered breathing |  |  |  |
|  | - Excessive daytime somnolence |  |  |  |
| **A** | Autonomic symptoms (A) |  |  |  |
|  | - Bladder disturbance: urgency, frequency, or nocturia |  |  |  |
|  | - Orthostatic hypotension |  |  | A decrease in systolic blood pressure of 20 mmHg or a decrease in diastolic blood pressure of 10 mmHg within three minutes of standing when compared with blood pressure from the supine position. |
| **GI** | Gastrointestinal symptoms (G) |  |  |  |
|  | - Constipation |  |  |  |
|  | - Nausea/vomiting |  |  |  |
|  | - Dysphagia and choking |  |  |  |
| MMSE: Mini-mental state examination, PDSS-2: Parkinson’s disease sleep scale 2^nd^ version, HAM-D: Hamilton Depression Rating Scale, HAM-A: Hamilton Anxiety Rating scale | | | | |

1.3) Gait and balance impairment (6 items; maximum score 12 points)

| **Location of the most severe symptoms** | **Gait and balance impairment** | **YES=1 NO=0** | **Multiply the most severe symptoms by 2** | **Remarks** |
| --- | --- | --- | --- | --- |
| **G** | Presence of falling in the past 1 month |  |  | UPDRS 13: Falling (unrelated to freezing)  YES: Presence of occasional falls, less than once per day |
|  | Freezing when walking |  |  | UPDRS 14: Freezing when walking  YES: Presence of occasional freezing when walking |
|  | Walking difficulty |  |  | UPDRS 15: Walking  YES: Presence of moderate difficulty, but requires little or no assistance |
|  | Gait impairment |  |  | UPDRS 29: Gait  YES: Presence at least of walking with difficulty, but requires little or no assistance; may have some festination, short steps, or propulsion |
| **P** | Postural instability |  |  | UPDRS 30: Postural stability (Retropulsion test or Pull test)  YES: Absence of postural response; would fall if not caught by examiner) |
|  | Difficulty arising from chair |  |  | UPDRS 27: Arising from chair  YES: Presence at least of pushing themselves up using the arms of the seat |
| YES; UPDRS scores for each item at least 2 out of 4 | | | | |

1.4) Co-morbidity (Select one item for weight problems; 5 items; maximum score 7 points)

| **Location of the most severe symptoms** | **Co-morbidity** | **YES=1 NO=0** | **Multiply score of the most severe symptoms by 2** | **Remarks** |
| --- | --- | --- | --- | --- |
| **V** | Visual impairment |  |  | YES: Presence of at least legally blind  (VA of 20/200 or less in the better eye with the best possible correction, and/or VF of 20 degrees or less) |
| **E** | Disabling hearing impairment |  |  | YES: Unable to hear and repeat words spoken in a normal voice at 1 meter |
| **J** | Knee and weight bearing joint problems |  |  |  |
| **W** | Morbid obesity |  |  | Extreme weight with BMI more than 40 kg/m2 |
|  | Significant weight loss |  |  | Significant weight loss is defined  as a loss of 4.5 kg or >5% of the usual body weight over a period of 6-12 months |
| VA; visual acuity, VF; visual field, BMI; body mass index | | | | |

1.5) Limitation of specific activities (7 items; maximum score 7 points)

| **Limitation on specific actions** | **YES=1 NO=0** | **Remarks** |
| --- | --- | --- |
| Walking up and down stairs |  | ABC-16; item 2 |
| Picking up slipper from the floor |  | ABC-16; item 3 |
| Reaching on tiptoes |  | ABC-16; item 5 |
| Standing on a chair to reach |  | ABC-16; item 6 |
| Getting in and out of the car |  | ABC-16; item 9 |
| Going up and down ramp |  | ABC-16; item 11 |
| Not holding escalator handrail |  | ABC-16; item 15 |
| ABC-16; activities-specific balance confidence scale-16 items, ABC-16 items 2,3,5,6,9,11,15 represent vertical related activities | | |

2) **Environmental component (total 112 points)**

2.1) Outdoor: outdoor area (9 items; maximum score 18 points)

| **Outdoor area** | **Problem with accessibility**  **(YES=1/NO=0)** | **Problem with usability**  **(YES=1/NO=0)** | **Injury as a result of the problem**  **(YES=1/NO=0)** | **Remarks** |
| --- | --- | --- | --- | --- |
| Path width |  |  |  | Narrow paths (≤ 0.9 meters) |
| Continuous path |  |  |  | Path or pavement laid with gaps wider than 5 mm |
| Walking surface |  |  |  | Unstable walking surface due to loose gravel, sand, clay, cracks, holes deeper than 5 millimeters, etc. |
| Entrance and routes with steps |  |  |  |  |
| Illogical route |  |  |  | Indirect pathway with no reason; creating confusion |
| Handrails on steep gradients |  |  |  | No handrails on steep gradients |
| Lighting |  |  |  | Less than 20 lux on working plane, 50 lux for night time |
| Warning on boundary or risk area |  |  |  | Step floor or boundary between two different floor surfaces without color contrast |
| Reliance on assisted device |  |  |  | Assisting person could be interpreted as an assisted device |
| Lux; a standardized unit of measurement of the light illuminance | | | | |

2.2) Outdoor: Entrance (6 items; maximum score 12 points)

| **Outdoor** | **Problem with accessibility**  **(YES=1/NO=0)** | **Problem with usability**  **(YES=1/NO=0)** | **Injury as a result of the problem**  **(YES=1/NO=0)** | **Remarks** |
| --- | --- | --- | --- | --- |
| **Home access** | | | | |
| Door openings and maneuvering space |  |  |  | Narrow door opening less than 0.90m |
| Door type |  |  |  | Swing door obstructing accessibility |
| Door weights |  |  |  | Heavy doors without automatic opening |
| Door knobs |  |  |  | Difficult to use/grasp |
| Door color |  |  |  | No color contrast between door pane and adjacent wall; and between handle and door pane |
| Door steps or doorsill |  |  |  | Presence of steps in front or under the doors |
| Lux; a standardized unit of measurement of the light illuminance | | | | |

2.3) Indoor: stairs (7 items; maximum 14 points)

| **Indoor** | **Problem with accessibility**  **(YES=1/NO=0)** | **Problem with usability**  **(YES=1/NO=0)** | **Injury as a result of the problem**  **(YES=1/NO=0)** | **Remarks** |
| --- | --- | --- | --- | --- |
| **Stairs** | | | | |
| Inappropriate location |  |  |  |  |
| Riser height |  |  |  | Inappropriate height of risers (lower than 13cm or higher than 15cm) |
| Stair treads |  |  |  | Inappropriate or irregular width (narrower than 30cm) |
| Handrails |  |  |  | No handrails on one or both sides of the staircase |
| Stair nose |  |  |  | Protruding part of stair nose is longer than 2cm |
| Lighting |  |  |  | Non-uniform and/or inadequate light illuminance (less than 100 lux) |
| Warning on boundary or risk area |  |  |  | No contrast on stair treads (for walking down the stair) |
| Lux; a standardized unit of measurement of the light illuminance | | | | |

2.4) Indoor: living room (7 items; maximum score 14 points)

| **Indoor** | **Problem with accessibility**  **(YES=1/NO=0)** | **Problem with usability**  **(YES=1/NO=0)** | | **Injury as a result of the problem**  **(YES=1/NO=0)** | **Remarks** |
| --- | --- | --- | --- | --- | --- |
| **Living room** | | | | | |
| Service area |  |  |  | | Insufficient area because of furnishing/storage units |
| Ease of operation (cabinet, drawer storage etc.) |  |  |  | | Position and handles are difficult to use (either positioned to high; out of reach or non-ergonomic design) |
| Sitting area |  |  |  | | Unsuitable sitting area (height less than 42cm or more than 45cm) |
| Lighting |  |  |  | | Non-uniform and/or inadequate light illuminance (less than 100 lux) |
| Light switch |  |  |  | | Light switch positioned lower than 70cm or higher than 100cm from floor level |
| Electrical Outlet |  |  |  | | Electrical outlet not positioned at 45-60cm |
| Path width |  |  |  | | Narrow paths ≤ 0.9 m |
| Lux; a standardized unit of measurement of the light illuminance | | | | | |

2.5) Indoor: kitchen (7 items; maximum score 14 points)

| **Indoor** | **Problem with accessibility**  **(YES=1/NO=0)** | **Problem with usability**  **(YES=1/NO=0)** | **Injury as a result of the problem**  **(YES=1/NO=0)** | **Remarks** |
| --- | --- | --- | --- | --- |
| **Kitchen** | | | | |
| Working space |  |  |  | Low working space (countertop height not in the range between 70 to 80cm and/or not accessible by chair or wheelchair) |
| Path Width |  |  |  | less than 0.9 m |
| Cupboards and shelves place |  |  |  | Inappropriate placement of cupboards and shelves; too high (higher than 170cm) or too deep |
| Size of controls and turning method |  |  |  | Very small or large, and complex maneuvers required |
| Slippery floor surface |  |  |  |  |
| Lighting |  |  |  | For overall space; less than 150 lux and for dining area; less than 200 lux |
| Reliance on assisted device |  |  |  | Assisting person could be interpreted as an assisted device |
| Lux; a standardized unit of measurement of the light illuminance | | | | |

2.6) Toilet/bathroom (12 items, maximum score 24 points)

| **Toilet/bathroom** | **Problem with accessibility**  **(YES=1/NO=0)** | **Problem with usability**  **(YES=1/NO=0)** | **Injury as a result of the problem**  **(YES=1/NO=0)** | **Remarks** |
| --- | --- | --- | --- | --- |
| Wet/dry area layout |  |  |  | The dry area is wet after shower |
| Space |  |  |  | Narrow paths ≤ 0.9 m |
| Grab bars in shower, bath, and/or toilet |  |  |  | - No grab bars in shower, bath, and/or toilet - Grab bars available but difficult to use |
| Shower seat |  |  |  | Height less than 42cm or more than 45cm |
| Wash-basin |  |  |  | Wash-basin height not in the range between 70 to 80cm and/or not accessible by chair or wheelchair |
| Toilet height |  |  |  | - Seat height less than 42cm or more than 45cm - Difficult to flush; use finger/pinch to operate flush - Difficult to use toilet paper holder and/or rinsing spray |
| Size of controls and turning methods |  |  |  | Very small or large, and complex maneuver required |
| Slippery floor surface |  |  |  | - Slippery floor surface  - Use floor mats |
| Path width |  |  |  | Less than 0.9 m |
| Lighting |  |  |  | Inadequate light illuminance (less than 100 lux) |
| Closet |  |  |  | Difficult to reach |
| Reliance on assisted device |  |  |  | Assisting person could be interpreted as an assisted device |
| Lux; a standardized unit of measurement of the light illuminance | | | | |

2.7) Bedroom (8 items; maximum score 16 points)

| **Bedroom** | **Problem with accessibility**  **(YES=1/NO=0)** | **Problem with usability**  **(YES=1/NO=0)** | **Injury as a result of the problem**  **(YES=1/NO=0)** | **Remarks** |
| --- | --- | --- | --- | --- |
| Service area |  |  |  | - - Pathway narrower than 90cm - - Furniture is not secured in its place |
| Handrails |  |  |  | No handrails installed at necessary location in the room |
| Environment for good sleep |  |  |  | With pollutants (noise and/or air) |
| Bed height |  |  |  | - Unsuitable height for bed (lower than 45cm or higher than 50cm) - No support bar to assist getting out of bed |
| Path width |  |  |  | Less than 0.9 m |
| Floor |  |  |  | Slippery floor |
| Lighting |  |  |  | - Scenario 1: sleep – more than 5 lux - Scenario 2: activity in bedroom – less than 100 lux |
| Reliance on assisted device |  |  |  | Assisting person could be interpreted as an assisted device |
| Lux; a standardized unit of measurement of the light illuminance | | | | |
