# Supplementary material for: Exploring Bedroom Usability and Accessibility in Parkinson’s Disease (PD): The Utility of a PD Home Safety Questionnaire and Implications for Adaptations
Source: Front Neurol. 2018 May 17;9:360. doi: 10.3389/fneur.2018.00360 (PMC5966531; doi:10.3389/fneur.2018.00360)
Supplement: Supplementary file 2 [file data_sheet_2.docx]

**Supplementary data 2:** Correlation analysis between personal and environmental components of the Parkinson’s disease home safety questionnaire

| **Correlation coefficient (r)** | Motor component | Gait-balance score | Underlying score | Limitation score | Indoor sum score |
| --- | --- | --- | --- | --- | --- |
| Total PD home safety score | - | - | 0.889*  (*p*=0.044) | 0.892*  (*p=*0.042) | - |
| Personal component score | 0.894*  (*p*=0.041) | 0.949*  (*p=*0.014) | - | - | - |
| Environmental component score | - | - | - | - | 0.975*  (*p*=0.005) |
| Spearman’s Rho correlation was used for correlation analysis. | | | | | |
